# When is an abscess more than an abscess? Syringe services programs and the harm reduction safety-net: a case report

**DOI:** 10.1186/s12954-020-00381-4

**Published:** 2020-06-01

**Authors:** Marcus Castillo, Margaret E. C. Ginoza, Tyler S. Bartholomew, David W. Forrest, Cestaki Greven, David P. Serota, Hansel E. Tookes

**Affiliations:** 1 Department of Medical Education, University of Miami Miller School of Medicine, Miami, FL USA; 2 Department of Public Health Sciences, University of Miami Miller School of Medicine, 1120 NW 14th St, Miami, FL 33136 USA; 3 Department of Anthropology, College of Arts and Sciences, University of Miami, Miami, FL USA; 4 IDEA Exchange, University of Miami Miller School of Medicine, Miami, FL USA; 5 Division of Infectious Diseases, Department of Medicine, University of Miami Miller School of Medicine, Miami, FL USA

**Keywords:** PWID, Syringe services program, SSTI, Abscess, Wound care, Student clinic

## Abstract

**Background:**

Syringe services programs (SSPs) are able to offer wrap-around services for people who inject drugs (PWID) and improve health outcomes.

**Case presentation:**

A 47-year-old man screened positive for a skin and soft tissue infection (SSTI) at an SSP and was referred to a weekly on-site student-run wound care clinic. He was evaluated by first- and third-year medical students, and volunteer attending physicians determined that the infection was too severe to be managed on site. Students escorted the patient to the emergency department, where he was diagnosed with a methicillin-resistant *Staphylococcus aureus* arm abscess as well as acute HIV infection.

**Conclusion:**

Student-run wound care clinics at SSPs, in conjunction with ongoing harm reduction measures, screenings, and treatment services, provide a safety-net of care for PWID and help mitigate the harms of injection drug use.

## Background

Injection drug use (IDU) continues to be a significant public health problem in the USA. High-risk injection practices, such as syringe sharing, have led to a secondary epidemic of infectious complications of drug use. This includes increasing IDU-associated infections from human immunodeficiency virus (HIV) [1, 2] and hepatitis C virus [3], as well as increasing hospitalizations for skin and soft tissue infections (SSTIs), osteomyelitis, septic arthritis, and endocarditis [4–9]. Establishment of syringe services programs (SSPs) is a proven harm reduction strategy that reduces HIV and HCV transmission among persons who inject drugs (PWID) [10].

SSPs are community-based prevention programs that provide PWID with access to sterile injection equipment (i.e., syringes, cookers, cottons, water). Beyond safer injection resources, SSPs provide PWID with a range of services, including HIV/HCV screening, prevention, and treatment [11–13]; linkage to substance use treatment [14, 15]; on-site treatment with medications for opioid use disorder (MOUD) [16]; overdose prevention through naloxone distribution [17]; and general medical services (i.e., wound care, primary care) [18]. More importantly, SSPs have been shown to be a cost-effective strategy to prevent HIV [19–22]. We report a case of undiagnosed acute HIV infection presenting as vague constitutional symptoms in the setting of a developing abscess identified by an SSP.

## Case presentation

The patient is a 47-year-old man with severe opioid use disorder who presented to the Infectious Disease Elimination Act (IDEA) SSP at the University of Miami Miller School of Medicine with right arm swelling and pain progressing over 3 days. On this day, he was visiting the student-run walk-in wound care clinic, which provides free medical services to a population with substance use disorders and often experiencing homelessness [23]. He was assessed in the clinic by a first-year medical student and a third-year medical student who reported that his right arm had become red, hot, swollen, and painful in the area where he normally injects fentanyl. The pain started in the forearm but had crossed into the dorsum of his hand impeding flexion at the wrist. On review of systems, the medical students elicited symptoms of fevers, night sweats, and 5 kg of unintentional weight loss over the past 2 weeks.

The medical students presented the case to the two volunteer attendings staffing the clinic—an internist and a surgeon—who then assessed the patient. Using a point of care ultrasound, they identified a large fluctuant abscess of the right forearm tracking over the wrist with a contiguous collection in the right hand. Diffuse cervical and axillary lymphadenopathy was noted on physical exam. The attendings determined that the infection was too severe to be managed with on-site incision and drainage and oral antibiotics. The students escorted the patient to the emergency department (ED) of the nearby county hospital with a written note from the IDEA clinic attending.

**Fig. 1:**
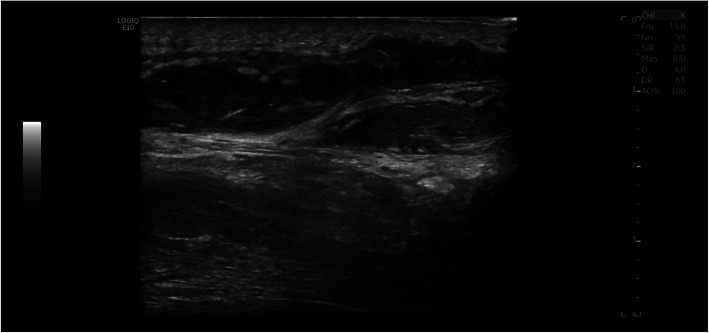
Right forearm dorsal medial sag

On routine opt-out screening labs in the ED, the patient tested reactive for HIV by 4th generation screening test with an indeterminate differentiation assay for HIV-1, suggesting acute HIV infection. HIV viral load by polymerase chain reaction returned at over 1 million copies/ml. Ultrasound of his right upper extremity in the ED showed a complex subcutaneous collection measuring 1.2 by 3.6 cm in axial dimension, associated with soft tissue edema (Fig. 1).

The patient was admitted to a teaching hospitalist service dedicated to the care of patients living with HIV, assessed by orthopedic surgery, and started on vancomycin, cefepime, hydromorphone, acetaminophen, and a nicotine patch. He initially had beside incision and drainage, but due to the severity of his SSTI, he was taken to the operating room for a more extensive debridement and irrigation of his abscess. Operative cultures ultimately grew methicillin-resistant *Staphylococcus aureus* (MRSA). He also initiated HIV antiretroviral therapy (ART) with an integrase inhibitor and two nucleoside reverse transcriptase inhibitors.

Antibiotics were optimized to intravenous vancomycin which was ultimately transitioned to oral doxycycline at the time of departure. The medical team planned to transition the patient to buprenorphine for his opioid use disorder, but on the sixth day of hospitalization, he left the hospital against medical advice, and presented to the IDEA SSP for evaluation and antibiotics. The IDEA team was able to convince him to return to the hospital, where he was successfully started on buprenorphine 8 mg twice daily.

Unfortunately, the patient was uncomfortable depending on MOUD to feel healthy and, as a result, stopped taking buprenorphine. He visits the IDEA SSP daily to obtain injection supplies, along with his ART and hepatitis C treatment, which are stored in the SSP’s pill lockers. Via the substance use disorder curriculum at the University of Miami, he is participating in small group discussions with medical students and sharing the benefits of harm reduction measures, the importance of safe injection practices, and his experiences with MOUD and stigma within the healthcare system. Even though he has discontinued MOUD, he keeps buprenorphine in his medication locker at the IDEA SSP so he can re-initiate when he is ready.

## Discussion and conclusions

PWID experience a multitude of complications due to injection drug use, and SSPs are well positioned to provide diagnostics, even for complicated cases. The SSP was a critical safety-net for the patient in this case, because shortly after the onset of symptoms, he presented to the SSP and the staff reminded him about the weekly student-run clinic. His comfort with the program, staff, and volunteer students facilitated his admission to the hospital. As he described in his own words:

Natural reaction for anybody who is an addict is usually just get out of there and go get high because it's an uncomfortable feeling… Because it was a real scary prospect. You know, you may be losing your arm, your hand or whatever. They stayed with me. They were really important in the whole process, because I don’t know how I would have reacted if it was under different circumstances.

Had the patient’s abscess gone untreated, the outcomes could have been far worse. Complications from SSTIs amongst PWID can lead to sepsis, infective endocarditis, and osteomyelitis. Early interventions on SSTIs reduce the risk of morbidity and mortality related to these invasive infections, and they have also been shown to decrease emergency department visits, surgical and inpatient admissions, and hospital expenses [24].

Up to a third of PWID globally have experienced an abscess within the past month [25]. Through early identification, SSTIs can often times be treated with antibiotics without requiring hospital visits or surgical procedures. However, stigma towards PWID can prevent them from seeking prompt medical care. Instead, up to a quarter of PWID with SSTIs have self-medicated, tried to self-lance their wounds, or sought out street procedures from untrained individuals. These attempts can occur in unsterile environments and lead to further complications of SSTIs [26].

For these reasons, it is essential to screen for SSTIs amongst PWID at SSPs. At the IDEA SSP, participants are asked a series of brief questions by the staff or student volunteers when they conduct syringe exchange, including whether the participant has experienced an SSTI due to injection drug use since their last visit to IDEA. Participants who indicate that they have an SSTI are reminded about the weekly student-run clinic. This non-judgmental space, staffed by students familiar to participants along with medical professionals, facilitates a continuum of care and improves outcomes for SSTIs. This low-barrier access to healthcare, including on-site provision of antibiotics and/or incision and drainage, is essential to mitigating the harms associated with injection drug use.

This patient ultimately stopped taking buprenorphine and resumed the use of injection opioids shortly after his hospital discharge. Following harm reduction philosophy’s tenet of respect for the individuals’ autonomy, IDEA SSP staff continued to support this patient. Despite the fact that the use of MOUD is associated with decreased opioid use and improved mortality, not all PWID desire to stop using drugs. This case demonstrates that abstinence from drug use should not be a prerequisite for receipt of other important medical care. The patient has continued to take his ART and HCV treatment with good adherence and continues to use drugs as safely as possible, equipped with supplies and support from the IDEA SSP. The non-stigmatizing approach and acceptance of the SSP facilitated linkage to the medical system that is often out-of-reach to the most vulnerable members of our community.

A safety-net of care with wrap-around services at an SSP is an effective way to prevent the progression and spread of illnesses among PWID. Routine HIV screening and rapid linkage to care at the IDEA SSP helped to prevent an HIV outbreak in Miami after the identification of ten anonymous HIV seroconversions in 2018 [27]. In the case of this patient, a routine screening for SSTIs at the wound care clinic prevented further complications of his abscess and led to his early HIV diagnosis, initiation of ART, and subsequent rapid viral suppression. For illnesses that cannot be diagnosed by simple screening tests, thorough histories and physical exams performed by student and physician volunteers at on-site clinics provide an opportunity for high-quality, comprehensive healthcare in a low-barrier setting. This safety-net of care helps to meet patients’ needs where they are, no matter the stage of their disease progression or recovery, thereby preventing life-threatening complications.

## Data Availability

Not applicable
