# Correction to: When is an abscess more than an abscess? Syringe services programs and the harm reduction safety-net: a case report

**DOI:** 10.1186/s12954-020-00390-3

**Published:** 2020-06-24

**Authors:** Marcus Castillo, Margaret E. C. Ginoza, Tyler S. Bartholomew, David W. Forrest, Costaki Greven, David P. Serota, Hansel E. Tookes

**Affiliations:** 1 Department of Medical Education, University of Miami Miller School of Medicine, Miami, FL USA; 2 Department of Public Health Sciences, University of Miami Miller School of Medicine, 1120 NW 14th St, Miami, FL 33136 USA; 3 Department of Anthropology, College of Arts and Sciences, University of Miami, Miami, FL USA; 4 IDEA Exchange, University of Miami Miller School of Medicine, Miami, FL USA; 5 Division of Infectious Diseases, Department of Medicine, University of Miami Miller School of Medicine, Miami, FL USA

**Correction to: Harm Reduction Journal (2020) 17:34**

**https://doi.org/10.1186/s12954-020-00381-4**

After publication of the original article [1], the authors identified an error in the author name of Costaki Greven.

The incorrect author name is: Cestaki Greven

The correct author name is: Costaki Greven

The author group has been updated above and the original article [1] has been corrected.
